# Carbon Sources for Polyhydroxyalkanoates and an Integrated Biorefinery

**DOI:** 10.3390/ijms17071157

**Published:** 2016-07-19

**Authors:** Guozhan Jiang, David J. Hill, Marek Kowalczuk, Brian Johnston, Grazyna Adamus, Victor Irorere, Iza Radecka

**Affiliations:** 1 School of Biology Chemistry and Forensic Science, University of Wolverhampton, Wolverhampton WV1 1LY, UK; Guozhan.jiang@wlv.ac.uk (G.J.); D.Hill@wlv.ac.uk (D.J.H.); M.Kowalczuk@wlv.ac.uk (M.K.); B.Johnston@wlv.ac.uk (B.J.); V.Irorere@wlv.ac.uk (V.I.); 2 Polish Academy of Sciences, Centre of Polymer and Carbon Materials, Zabrze 41-819, Poland; Grazyna.Adamus@cmpw-pan.edu.pl

**Keywords:** polyhydroxyalkanoates, microorganisms, carbon sources, biorefinery

## Abstract

Polyhydroxyalkanoates (PHAs) are a group of bioplastics that have a wide range of applications. Extensive progress has been made in our understanding of PHAs’ biosynthesis, and currently, it is possible to engineer bacterial strains to produce PHAs with desired properties. The substrates for the fermentative production of PHAs are primarily derived from food-based carbon sources, raising concerns over the sustainability of their production in terms of their impact on food prices. This paper gives an overview of the current carbon sources used for PHA production and the methods used to transform these sources into fermentable forms. This allows us to identify the opportunities and restraints linked to future sustainable PHA production. Hemicellulose hydrolysates and crude glycerol are identified as two promising carbon sources for a sustainable production of PHAs. Hemicellulose hydrolysates and crude glycerol can be produced on a large scale during various second generation biofuels’ production. An integration of PHA production within a modern biorefinery is therefore proposed to produce biofuels and bioplastics simultaneously. This will create the potential to offset the production cost of biofuels and reduce the overall production cost of PHAs.

## 1. Introduction

Polyhydroxyalkanoates (PHAs) are a group of bacterial polyesters produced by a variety of prokaryotic microorganisms under unbalanced nutrition conditions as carbon and energy storage materials [1]. PHAs, due to their superior biodegradability, biocompatibility and thermal processability [2,3], are currently produced by many companies, such as Metabolix^®^ (Woburn, MA, USA), Procter & Gamble Co., Ltd. (Cincinnati, OH, USA), Tianjin Green Bioscience Co., Ltd. (Tianjin, China) and Biocycle PHB Industrial SA (Serrano, SP, Brazil) [2]. PHA production capacity has increased rapidly over the last decade, which according to statistics from the European Bioplastics Association, reached up to 34,000 tonnes of global production capacity in 2013 [4]. The U.K. share of this potential market is currently £2 million [5].

However, the expanding PHA industry has raised concerns, since most PHAs are produced primarily from food crops, sugar cane and vegetable oils, as listed in Table 1. Production of these carbon sources competes with food supply production. For example, when corn is used as the carbon source for substrate glucose, one kilogram of corn can produce 0.67 kg of glucose [6], and this amount of glucose can produce 0.27 kg of PHAs [7]. Consequently, 34,000 tonnes of PHAs would utilise about 126,000 tonnes of corn. Therefore, it is essential to exploit non-food-based carbon sources for a sustainable production.

**Figure 1:**
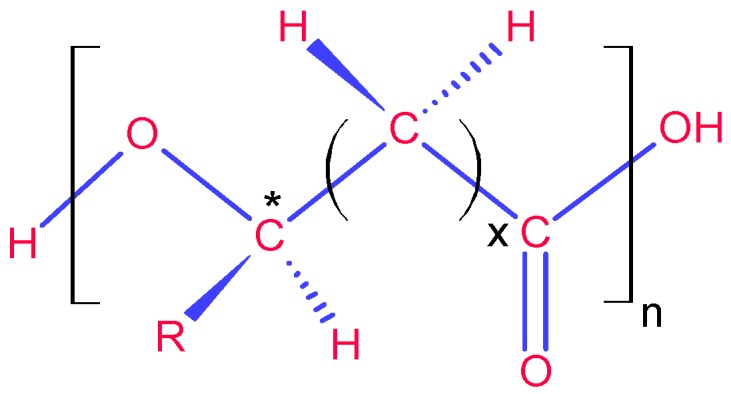
The general structure of a polyhydroxyalkanoate (PHA) macromolecule. All monomeric units have one chiral centre (*) with the configuration of the *R* enantiomer, and the whole macromolecule has a perfect isotactic structure [12]. The number of carbon atoms in the *R* substituent ranges up to 18 [13]; *x* ranges from 1–3, and *n* ranges from 100–30,000 [3,14]. For poly(3-hydroxybutyrate) (P3HB), *R* = CH_3_ and *x* = 1; for poly(3-hydroxyvalerate) (P3HV), *R* = C_2_H_5_ and *x* = 1; etc.

In this paper, we will review PHA production in terms of substrates and the derivation processes of the substrates from various carbon sources. The aim of this study is to identify opportunities and constraints related to the future sustainable production and to assess the framework for the implementation of the production within a biorefinery. For convenience in later discussions, the structure of PHAs is shown in Figure 1. These structurally-diverse polymers are composed of hydroxyalkanoic units with different numbers of carbon atoms by varying *R* and *x*. In the following text, the abbreviations of the names of PHAs are used.

## 2. Overview of Substrates for Polyhydroxyalkanoates (PHAs) Production

**Figure 2:**
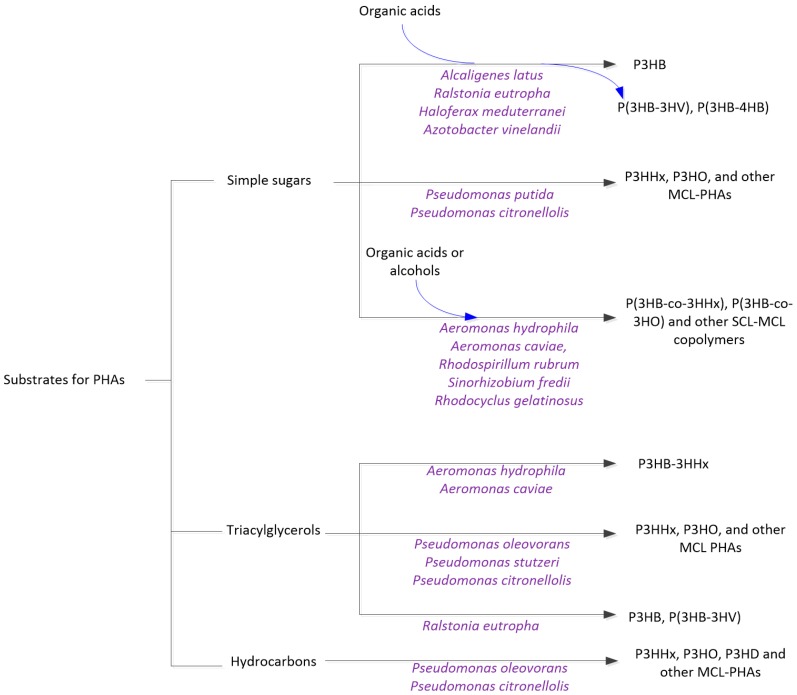
A summary of the substrates and the microorganisms that can synthesize PHAs. P3HHx = poly(3-hydroxylhexanoate); P3HO = poly(3-hydroxyoctoate); P3HD = poly(3-hydroxydodecanoate); P3HB = poly(3-hydroxybutyrate); P3HV = poly(3-hydroxyvalerate). A “-co-“ is used to indicate the copolymer. For example, P(3HB-co-4HB) is the copolymer of 3-hydroxybutyrate and 4-hydroxybutyrate. For other abbreviations, refer to Figure 1. Blue arrows indicate substrates added later in the fermentation process to make copolymers.

PHAs are synthesized in bacterial cells through a metabolic process. The substrates for biosynthesizing PHAs are usually restricted to small molecules, since bacteria have thick, rigid cell walls surrounding membranes. Large polymeric molecules cannot be transported into the cell, and an extracellular transformation either by the microorganism or by a chemical process is needed for the use of the polymeric molecules. A summary of the substrates for PHA synthesis and typical microorganisms that can use the substrates is given in Figure 2.

As shown in Figure 2, the substrates can be generally divided into three categories: simple sugars (monosaccharides), triacylglycerol and hydrocarbons. Most PHA-producing microorganisms can use simple sugars, whilst triacylglycerol have only been reported for some microorganisms. Hydrocarbon metabolism is more limited, but can be used by *Pseudomonas* species of bacteria. For the same substrate, different bacteria can produce PHAs with a different composition. For example, *Pseudomonas* spp. can use glucose and other simple sugars to synthesize medium chain-length PHAs, such as poly(3-hydroxylhexanoate) (P3HHx) [15,16,17], while *Ralstonia eutropha* synthesizes only poly(3-hydroxybutyrate) (P3HB) using glucose [18].

To better understand the conversion of various substrates to PHAs in Figure 2, one can refer to the catalytic conversion of substrates in a chemical process. In the biosynthesis of PHAs, the bacterial cells can be regarded as biochemical catalysts, carrying out the metabolic reactions to transform the substrates into different PHAs. Just like chemical catalysts have selectivity to substrates and the same substrate can be converted into different products by using varying catalysts, the same is true for PHA-producing bacteria due to their substrate specificity [2].

Organic acids and alcohols (Figure 2) are used as precursors for biosynthesis of copolymers. For example, propionic acid is added at late stages of fermentation to produce poly(3-hydroxybutyrate-co-3-hydroxyvalerate) (P(3HB-co-3HV) copolymer when glucose is used as the main substrate [19].

The main growth substrates come from various carbon sources after the appropriate transformation is applied. The cost of the carbon source accounts for approximately 50% of the entire production process of PHAs [20]. Simple sugar substrates are mainly derived from carbohydrates; triacylglycerols are the main components of oils from plants and fats from animals; a potential source of fermentable hydrocarbons is waste plastics. In the following, the use of these carbon sources and their transformation processes are reviewed, placing an emphasis on the sustainability in order to provide the best vantage point to identify sustainable carbon sources for PHA production.

## 3. Carbohydrates

**Figure 3:**
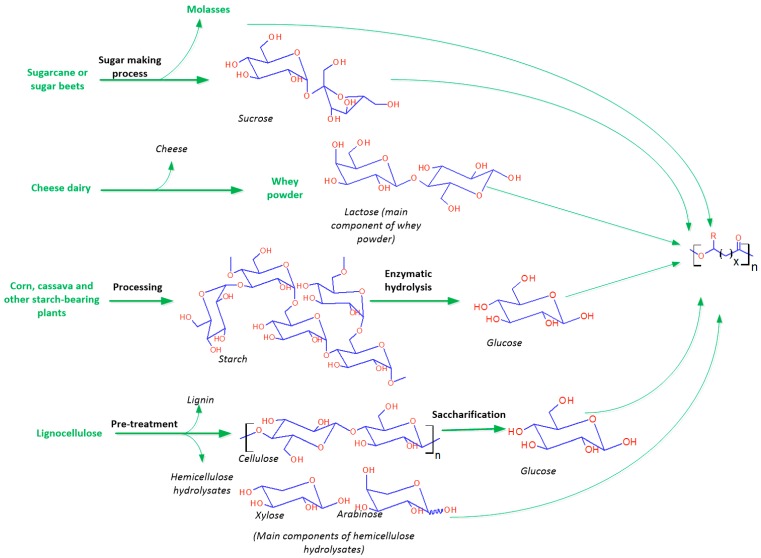
Carbohydrate carbon sources and their transformation processes to simple sugars for fermentative production of PHA. Materials in green indicate the starting raw material from which microbial growth subtrates (in black) are derived.

Carbohydrates can be classified into monosaccharides, oligosaccharides and polysaccharides and can be hydrolysed to simple sugars (monosaccharides). Polysaccharides are carbohydrates in polymerised form, including mainly starch, cellulose and hemicellulose. Monosaccharides and disaccharides can be fermented directly to produce PHAs, while polysaccharides are not fermentable unless hydrolysed first. Figure 3 summarizes the derivation of fermentable simple sugars from plants.

### 3.1. Sucrose

Sucrose is sourced from sugar-bearing raw materials, such as sugar beet in temperate climates and sugar cane in tropical regions. Sucrose is composed of a glucose unit linked to a fructose via a glycoside linkage. *Ralstonia eutropha*, which has been extensively used for PHA production, cannot use sucrose directly [21]. However, the following wild-type bacterial strains have been identified to produce PHAs from sucrose: *Azotobacter vinelandii* [22], *Alcaligenes latus* [23] and *Hydrogenophaga pseudoflava* [24]. These microorganisms can hydrolyse sucrose extracellularly in the early stage of cultivation into glucose and fructose, and both are subsequently used for cell growth [25]. The optimised culture conditions of *Alcaligenes latus* with sucrose was determined by Grothe et al. [23]: 25–37 °C, ammonium chloride or ammonium sulphate as the nitrogen source and a pH value of 6.5. Under these conditions, P3HB was produced and constituted up to 63% of dry cell weight with the average PHA yield in the region of 0.40 g/g sucrose.

Recombinant microorganisms have also been investigated for direct use of sucrose. For example, Zhang et al. [26] harboured PHA synthesis genes from *Ralstonia eutropha* in *Klebsiella aerogenes*. A commensurate result with wild-type bacteria was achieved in a 10-L batch fermenter. However, the recombinant bacteria exhibited instability. Park et al. [21] introduced sucrose-utilizing genes of *Mannheimia succiniciproducens* into *Ralstonia eutropha* enabling it to produce PHAs from sucrose. The recombinant *R. eutropha* when grown in a 5-L batch fermentor produced an average of 0.0046 g·L^−1^·h^−1^ of PHA, a relatively very low productivity for PHA production.

An inexpensive and sustainable source of sucrose for producing PHA is molasses. During the production of sucrose from sugarcane or sugar beets, a large amount of viscous molasses is produced as a by-product, which has a high sucrose content. The use of the sucrose contained in molasses for producing PHAs was first developed by Page [22,27,28] using *Azotobacter vinelandii* for the fermentation of beet molasses. After 40 h by fed-batch culture, the yield of PHA was 23 g·L^−1^ with 66% PHA of cell dry weight. The productivity was 0.575 g·L^−1^·h^−1^, and the yield was 0.12 g PHA per gram of molasses consumed. Molasses has been widely used as a carbon source in industrial scale fermentations due to their relatively low price and abundance. However, the extent of the availability of molasses is insufficient to satisfy the future increasing demand of PHA production.

### 3.2. Lactose

Lactose is a disaccharide consisting of galactose and glucose via a glycoside linkage. An inexpensive and sustainable source of lactose is whey, the by-product of cheese and casein production. Whey permeate contains 35–50 g·L^−1^ of lactose depending on how the milk has been curdled [29]. The following bacterial strains have been identified to synthesize PHAs directly from whey or whey permeate: *Hydrogenophaga pseudoflava* DSM 1034 and *Sinorhizobium meliloti* 41 [30,31]; recombinant *Escherichia coli* [32,33]; *Methylobacterium* sp. ZP24 [34]; *Thermos thermophilus* HB8 [35]; *Bacillus megaterium* [36]. Ahn et al. [32] used a recombinant strain of *E. coli* harbouring *Alcaligenes latus* PHA biosynthase genes to produce PHAs with lactose in a fed-batch mode. A productivity of 2.57 g PHB L^−1^·h^−1^ was achieved. However, the PHAs produced using *H. pseudoflava* contained 3-hydroxyvalerate (3-HV) and 4-hydroxybutyrate (4-HB) units [31].

Koller et al. [37] employed a two-step process to produce PHA from whey using *Pseudomonas hydrogenovora*, which cannot use lactose directly. First, whey permeate was enzymatically hydrolysed into monosaccharides, glucose and galactose, and then, the hydrolysate was used to produce PHA with *Pseudomonas hydrogenovora*. Both the yield and productivity of the two process are much lower than using the recombinant strains. Although lactose from whey can be used for PHA production, the extent of whey produced would only satisfy a small portion of even current total PHA production.

### 3.3. Starch

Starch is a polymer of d-glucose with α-1,4-glycosidic bonds. It is a main constituent of corn, rice, potato, cassava and sorghum. Under enzymatic or acid hydrolysis, starch is hydrolysed first into maltose and then glucose, which is the most extensively-used carbon source for industrial production of PHAs. The earliest commercial PHA was produced in the United Kingdom by Imperial Chemical Industries (ICI) using starch-derived glucose with *R. eutropha* [19]. This ICI technology requires an organic acid like propionic acid as the co-substrate for producing the desired copolymer; otherwise, only homopolymer P3HB is produced from glucose. The commercial production of P3HB using *Alcaligenes latus* by Chemie Linz also used starch as the starting substrate [38].

### 3.4. Lignocellulose

Since starch, sucrose and lactose are a major food source for humans and animals, derivation of glucose and other simple sugars from lignocelluloses has received great attention. Lignocellulose is the most abundant and sustainable carbon resource, which contains about 40%–50% cellulose, 20%–50% hemicelluloses and 20%–30% lignin. Lignin is not fermentable due to its complex aromatic nature [39,40]. However, cellulose and hemicellulose are valuable, sustainable source of fermentable sugars. Cellulose is composed of β-1,4-glycosidic bonds of d-glucose, which is neither soluble in water nor digestible by human beings due to its large molecular weight and crystalline structure. Hemicellulose is an amorphous polysaccharide of several simple pentoses, such as xylose, mannose, arabinose, galactose and rhamnose, with xylose is often the major component.

The first step in the use of lignocellulose is delignification, in which cellulose and hemicelluloses are separated from the lignin. The separation methods include dilute acid hydrolysis [41], steam-explosion, hydrothermal treatment, lime treatment and ammonia treatment [42]. After these processes, the following three components are produced: hemicellulose hydrolysate, cellulose and lignin.

The hydrolysates from hemicellulose are mainly composed of pentoses, such as xylose and arabinose, but also contain acetic acid, acid soluble lignin, small amount of alcohols, volatile acids, small amount of hexose and dehydrated hexose and pentose [43]. The pure forms of these pentoses are fermentable to PHAs. Bertrand et al. [44] compared the fermentation of xylose and arabinose with fermentation of glucose using *Pseudomonas pseudoflava*. Xylose and arabinose as substrates had a conversion of 0.17–0.19 g PHA/g, which was much lower than glucose as a substrate, which had a conversion of approximately 0.40 g PHA/g. Moreover, the productivity of the PHA using xylose and arabinose was four- to five-times slower than when the cells used glucose. The molecular weight of P3HB from xylose fermentation is comparable to that from glucose, but the molecular weight of the P3HB from arabinose fermentation was only half of that of PHA from glucose. This differences in molecular weight, however, would not significantly affect the mechanical performance of the PHA materials. Young et al. [45] and Ramsay et al. [46] showed that *Pseudomonas cepacia* was better than *P. pseudoflava* for xylose fermentation. The maximum specific growth rates of cells on xylose and on glucose were similar. Batch fermentation of 30 g·L^−1^ of d-xylose with nitrogen limitation by *P. cepacia* produced 48%–56% by weight of P3HB per cell dry weight, and the yield of P3HB was 0.11g PHB/g xylose. Lee et al. [47] demonstrated that *E. coli* harbouring PHA synthesis genes of Ralstonia eutropha was able to accumulate PHB from xylose up to 74% of cell dry weight with a yield of 0.226 g PHB/g xylose, which is better than wild-types. More recently, Lopes et al. [48] screened bacteria on xylose for PHA production and attained a maximum yield of 0.26 g PHA/g xylose.

Direct use of the hydrolysates of hemicelluloses as a mixture of sugars has also been attempted. At high concentrations, the dehydrated products of the sugars and acid components in hemicellulose hydrolysates are inhibitors for bacterial growth. However, at low concentrations, these inhibitors can be used by *R. eutropha* mainly for cell growth as indicated by Yu et al. [49]. In order to use the mixture as a whole, fed-batch fermentation would be preferred, since organism growth and PHA production in batch cultures would be affected by the inhibitory substances in the raw material, whereas in the fed-batch culture, they could be added at their rate of utilisation [46].

Cellulose can be used to produce glucose via saccharification, and the produced glucose is mostly used to produce the second generation ethanol; however, attempts have also been made to produce PHAs using cellulose-derived glucose. Traditional hydrolysis contains dehydration products of glucose, which are inhibitory to most PHA-accumulating microorganisms [50]. An efficient hydrolysis of cellulose via Ru (ruthenium) or Pt (platinum) catalyst can provide glucose as the major product containing only a small amount of the dehydrated product of hexose (hydroxylmethylfuran) [51]. Furthermore, a hydroxylmethylfurfuran-resistant engineered *E. coli* is able to produce P3HB with this cellulose hydrolysate [52]. It was found that after 60 h of fermentation, the cell dry weight reached 5.6 g·L^−1^ with a polymer content of 59%. In contrast, when pure glucose was used as the substrate, the cell dry weight was 5.9 g·L^−1^ with a polymer content of 58%. Recently, a novel saccharification involved the use of ionic liquids to improve the yield of fermentable sugars [53].

Fermentation of hydrolysates of the mixture of cellulose and hemicelluloses is a promising approach to produce PHA from lignocelluloses. Cesario et al. [54] conducted a fermentation of the hydrolysates of wheat straw using *Burkholderia sacchari*. The mixture contained mainly glucose, xylose and arabinose. *B. sacchari* was grown on the mixture, accumulating about 60% of cell dry weight and a conversion of 0.19 g P3HB/g. In comparison, fermentation of a mixture of commercial hexose and pentose sugars yielded about 70% of cell dry weight of PHAs with a yield of polymer of 0.18 g/g.

Another interesting example is the use of liquefied wood as an inexpensive co-substrate with glucose to provide 3-hydroxyvalerate units in the produced PHAs [55]. This is because liquefied wood without further pre-treatment is much less expensive than the commercially-used co-substrates, such as pentanoic acid or propanoic acid. It is acknowledged that the levulinic acid derived from the cellulose component in wood is transformed into a 3-hydroxyvalerate (3HV) component. Koller et al. [55] demonstrated this process during growth of *Cupriavidus necator* in a 7.5-L fermenter in batch mode. When compared to fermentation with glucose only, both the productivity of PHA (2.84 g·L^−1^·h^−1^) and the molar mass of the resulting PHA (3.52 × 10^5^) increased.

## 4. Triacylglycerols

### 4.1. Direct Fermentation

**Figure 4:**
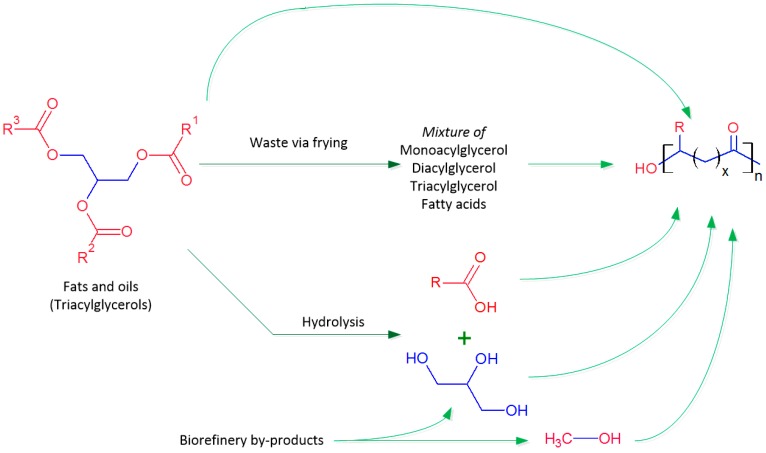
Fats and oils as substrates for PHA production via fermentation of various microorganisms.

Triacylglycerols are the main components of animal fats and plant oils. In the molecules of triacylglycerols, three fatty acids are attached to a glycerol backbone. Figure 4 shows the structures and routes for the use of triacylglycerols for producing PHAs. Triacylglycerols containing only saturated long-chain fatty acyl groups tend to be solid (fats) at body temperature, and those containing unsaturated or short-chain fatty acyl groups tend to be liquid (oils) [56].

Direct use of triacylglycerols requires triacylglycerol-utilising bacteria, which can secrete lipases [57]. The released lipases in the fermentation media catalyse the release of fatty acids from triacylglycerol molecules [58]. The fatty acids are then transported into the cell through the cell membrane, where they are catabolised via a β-oxidation cycle to produce PHA monomers and then synthesized into PHAs.

#### 4.1.1. Animal Fats

Waste animal fats from food processing and slaughtering industries have a huge potential as the carbon source for PHA production. However, due to their high melting temperature, waste animal fats can be problematic during fermentation processes. Cromwick et al. [59] tested four *Pseudomonas* sp. (*Pseudomonas oleovorans*, *Pseudomonas resinovorans*, *Pseudomonas putida* and *Pseudomonas citronellolis*) with tallow in shake flask cultures. They found that only *P. resinovorans* could produce PHA polymer on unhydrolyzed tallow, with a PHA content of 15% of the cell dry weight. However, the ester of the fatty acids from tallow with methanol can be fermented using *P. citronellolis*, with a productivity for medium chain length (mcl)PHA of 0.036–0.050 g·L^−1^·h^−1^ and PHA contents of 20.1%–26.6% (wt) [60]. Later, Taniguchi et al. [61] used *R. eutropha* to ferment animal fats in a shake flask culture. They achieved 80% P3HB in the dry cell weight with a 1% 3HV unit.

Recently, Riedel et al. [62] conducted a fermentation in a 5-L fermentor of tallow with *R. eutropha.* In their experiments, gum arabic was used as the emulsifying agent to generate a homogeneous state. In a fed-batch mode, the dry cell weight and PHA content were 45 and 26 g/L, respectively, with a yield of 0.40 g PHA/g fat and a productivity of 0.36 g·L^−1^·h^−1^.

#### 4.1.2. Plant Oils

Plant oils are relatively easy to ferment due to their liquid form. The first demonstration of using triacylglycerol as a carbon source for PHA synthesis was conducted by Shiotani and Kobayashi in 1993 with *Aeromonas caviae* [63]. The fermentation produced complex copolymers when the bacteria were grown on olive oil. In shake flask cultures, *Chromobacterium* sp. was able to yield P3HB with a content of about 50% with various plant oils, and the produced P3HB had a molecular weight range of 2 × 10^5^–6 × 10^5^ [64]. An advantage of using this bacterial strain was the high conversion of olive oil up to 0.88 g/g, which is much higher than *Aeromonas caviae* and *R. eutropha*. *R. eutropha* H16 was able to produce P3HB homopolymer up to approximately 80% (*w*/*w*) of the dry cell weight during its stationary growth with various plant oils [65].

A recombinant strain of *R. eutropha* (DSM 541, a PHA-negative mutant) harbouring a PHA synthase gene from *A. caviae* was also tested for fermenting plant oils [65]. Copolymer P(3HB-co-3HHx) with 4%–5% of 3-HHx was produced from these plant oils with approximately 80% PHA of the dry cell weight. When the recombinant *R. eutropha* was used in a 10-L fermentor with 20 g·L^−1^ of soybean oil as the sole carbon source, similar results were achieved [66]. The recombinant strain is now in use commercially by Procter & Gamble Co., Ltd. (Cincinnati, OH, USA), to produce (P(3HB-co-3HHx) [2].

### 4.2. Fatty Acids

There are many bacterial strains that can be grown on long-chain fatty acids, such as *Alcaligenes* AK 201 [67] and *Pseudomonas* sp. [59]. mcl-PHAs are produced from fatty acids by Pseudomonads [68], while *Alcaligenes* sp. produces short chain length (scl)-PHAs. Long chain fatty acids do not show any toxic effect on bacteria, while short chain organic acids with 3–5 carbons usually inhibit bacterial growth [69]. Therefore, only a low concentration of short chain organic acids (1–4 g·L^−1^) should be used for the bacterial production of P(3HB-co-3HV) [70].

### 4.3. Glycerol

Currently, one of the major uses of fats and oils is in the production of biodiesel by transesterification with methanol. Glycerol is the by-product of this process [71]. With every 100 tons of biodiesel produced via transesterification of vegetable oils or animal fats, about 10 tons of crude glycerol is produced [72]. Conversion of the crude glycerol into PHAs is a promising route to offset the production cost of biodiesel and to valorise the crude glycerol. When the crude glycerol is used for fermentation, it should be borne in mind that the crude glycerol contains mono-/di-/tri-glycerides, soap, methanol and salts as impurities [73].

Fermentative production of PHAs from glycerol has been investigated in recent years. Cavalheiro et al. [74,75] described a process for direct fermentation of glycerol using *Cupriavidus necator* (*R. eutropha*) and achieved a maximum cell dry weight of 82.5 g·L^−1^; the productivity was approximately 0.6–1.5 g·L^−1^·h^−1^, and the P3HB content was 62% for pure glycerol. When crude glycerol from biodiesel production was used, these values were lower. The maximum cell dry weight was 68.8 g·L^−1^, with a productivity of 0.84 g·L^−1^·h^−1^ and a P(3HB) content of 38%. Zhu et al. [76] conducted a 400-L pilot plant-scale fermentation of crude glycerol with *P. cepacia* using a fed-batch process. After 120 h of fermentation, the yield of dry biomass was 23.6 g·L^−1^, of which 7.4 g·L^−1^ was P3HB. The P3HB produced had a molecular weight from 50 to 3000 kDa. Although other strains have also been attempted for glycerol fermentation, such as recombinant *E. coli* [77], *Pseudomonas corrugata* and *P. oleovorans* [78], the PHA content and dry cell weight were low, while the polymers produced were the copolymers of 3-hydroxybutyric acid and other medium chain-length hydroxyalkanoic acids [79].

In using both *R. eutropha* and *P. cepacia*, the concentration of glycerol must be kept as low as 3% (*w*/*v*). At higher concentrations of glycerol, the specific growth rate of the strains, P3HB content and the molecular weight all decrease, which may be attributed to high osmotic stress, low enzymatic efficiency and earlier termination by glycerol [76].

### 4.4. Methanol

Methanol is the raw material for producing biodiesel by transesterification with fats and oils, and consequently, the waste streams of biodiesel production contains methanol. Methylobacterium sp. are capable of growing on one-carbon compounds, such as methanol [80]. Kim et al. [81] used methanol to produce P3HB under potassium limitation with *Methylobacterium organophilum*. At optimal conditions, the volumetric productivity reached 1.8–2.0 g P3HB L^−1^·h^−1^, and the yield was 0.19 g/g methanol. The concentration of methanol should be controlled below 0.5% (*v*/*v*) to prevent the inhibition of the growth of the strains [80,81]. On the other hand, the presence of methanol in crude glycerol can significantly suppress the growth of *R. eutropha* [82].

### 4.5. Waste Frying Oil

An inexpensive sustainable source of plant oils for PHA production is waste frying oil. After numerous uses, the oils are discarded as industrial waste. The oils undergo several chemical reactions, such as hydrolysis, thermal oxidation and polymerisation when repeatedly exposed for a long time at elevated temperatures. Waste frying oil generally consists of 70% triacylglycerol, while the remaining fraction consists of oil degradation products, which includes a range of new polar compounds, such as oligomeric or polymeric triacylglycerol, diacylglycerol, monoacyl-glycerol, free fatty acids, aldehydes and ketones [83].

Utilisation of waste frying oil for producing PHA has been successfully demonstrated. Obruca et al. [84] used waste rapeseed oil and propanol as a co-substrate with *R. eutropha* to produce P(3HB-co-3HV). Verlinden et al. [85] conducted similar research using waste rapeseed oil for producing PHAs with the same strain. In both investigations, it is interesting to note that waste frying oil enhanced the production of PHA because of other nutrients present in it. In a 5-L batch fermentation and using *R. eutropha*, Morais et al. [86] achieved a productivity of 0.14 g PHB L^−1^·h^−1^ and a yield of 0.14 g PHB/g waste oil.

## 5. Hydrocarbons

### 5.1. Fermentation of Hydrocarbons

Hydrocarbons can be metabolised by many microorganisms. Both gaseous n-alkanes and 1-alkenes (C1–C6) and long-chain paraffinic or olefinic hydrocarbons up to 44 carbons can be used by these microorganisms [87]. However, not all of these hydrocarbon-utilising microorganisms can accumulate PHAs. de Smet et al. [88] first identified that *P. oleovorans* and other fluorescent Pseudomonads can be grown on octane and accumulate mcl-PHAs in 1983. Later, Brandl et al. [68] investigated in detail the use of *P. oleovorans* to produce PHAs from n-alkanoic acids and found that octanoate and nonanoate gave maximum isolated polymer yields of approximately 30% of the cell dry weight. Chayabutra and Ju [89] grew *Pseudomonas aeruginosa* on hexadecane and found that PHA synthesis was compromised by the co-synthesis of a rhamnolipid. PHA synthesis was found to occur only during active cell growth, while substantial rhamnolipid production began during the onset of the stationary phase.

A number of *Pseudomonas* strains can also accumulate PHA from a variety of aromatic hydrocarbons [90,91], which is why pyrolysis oil of polyethylene terephthalate (PET) can be fermented to produce PHAs, as shown in the following section. There are, however some non-*Pseudomonas* species that can produce PHA from aromatics, e.g., *Rhodococcus aetherivorans* IAR1 [92]. One advantage of *Pseudomonas* strains is that they can produce very effective surfactants to solubilize/emulsify the insoluble hydrocarbons to facilitate their transportation into the cells through the hydrophobic cell walls.

The PHAs produced from hydrocarbons are characteristic of an alkyl group that varies from a propyl to a dodecyl group depending on the substrates used [89,93]. However, the PHA productivity during growth on hydrocarbons tends to be low, making these substrates an unlikely future choice for PHA production. 

### 5.2. Hydrocarbons Derived from Waste Plastics

**Figure 5:**
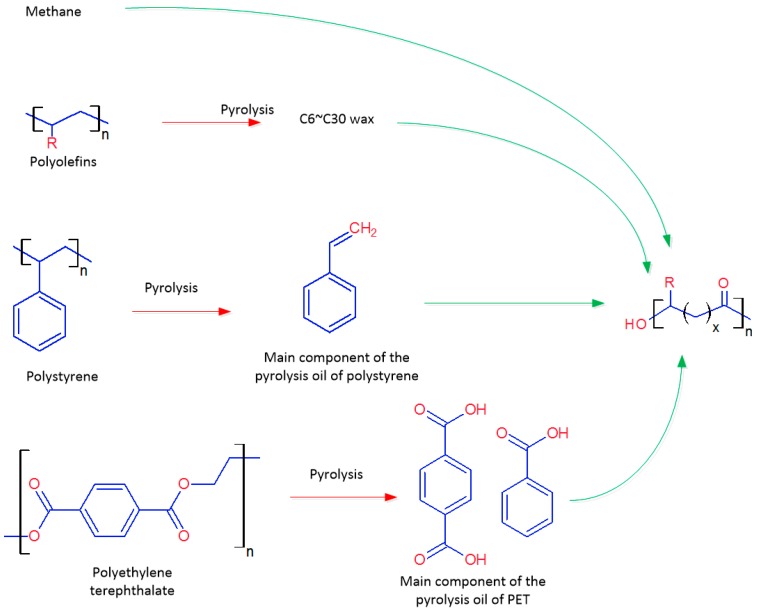
Hydrocarbons as substrates for PHA production and their derivation processes. Red and green arrows indicate chemical and biological processes respectively.

The use of hydrocarbons as substrates for producing PHAs is not of significant economic value. In recent years, hydrocarbons made from pyrolysis of waste plastics have been investigated. According to the European Association of Plastics Manufacturers, the global plastics production was 299 × 10^6^ tonnes in 2013 and will continue to grow for another 50 years. In Europe alone, 25.2 × 10^6^ tonnes of post-consumer plastic waste ended up in the waste upstream in 2012, among which 62% was recovered through recycling and energy recovery processes, while 38% still went to landfill. For energy recovery processes, plastics are usually pyrolysed to produce wax, which can be burned to generate energy in power plants or in ship engines. Types of waste plastics and their pyrolysis products that can be fermented to produce PHAs are shown in Figure 5. The waste plastics that can be used include: polyolefins, polystyrene and polyethylene terephthalate.

A pyrolysis process is used in a fluidized bed reactor for making hydrocarbon substrates. In the fluidized bed, fine quartz sand particles are fluidized at a temperature range of 450–550 °C by pre-heated nitrogen gas. The shredded plastic particles are then feed into the reactor via a screw feeder. The plastic particles are thermally decomposed into small hydrocarbon molecules, and then, the pyrolysis vapour leaves the reactor together with the fluidizing gas. The pyrolysis vapour is then condensed and collected as pyrolysis oil, which is in a semi-solid or liquid form dependent on the molecular mass.

Various pyrolysis oils and waxes have been investigated for producing PHA with hydrocarbon-utilising bacterial strains. Most of the produced PHAs are of medium chain length when *Pseudomonas* sp. are used for growth on styrene produced from the pyrolysis of polystyrene [94], polyethylene wax with a molecular weight of approximately 600 [95] and terephthalic acid for pyrolysis of PET [96]. *R. eutropha* has also been shown to accumulate PHAs using polyethylene wax as the carbon source [97], but the product is mainly P3HB with a minor proportion of other medium chain-length hydroxyalkanoic units. However, the majority of these fermentations are still at their preliminary stages. Much improvement is needed to increase both PHA content and productivity.

### 5.3. Methane

Another potential hydrocarbon substrate for PHA production is methane, which is readily available in oilfields and the degradation of organic matter biologically. Type II methylotrophs are known to produce P3HB from methane [20]. The theoretical yield of methane conversion to PHB is estimated at 67% using stoichiometric chemical equations [98]. However in reality, type II methanotrophic bacteria use up a large portion of their oxygen and methane to generate nicotinamide adenine dinucleotide phosphate (NADP^+^) (through the conversion of CO_2_) for acetoacetyl-CoA reductase and then P3HB production. With that in mind the conversion of methane to P3HB drops to 54% (without considering the effects of regenerated NADP^+^). In 2001, a yield of 0.55 g PHB g^−1^ CH_4_ and a productivity of 0.031 g PHB L^−1^·h^−1^ were reported using *Methylocystis* spp., which closely matched the theoretical predictions [99]. Recently, a high throughput microbioreactor system was developed to optimise medium composition for type II methylotrophs to produce PHAs. At optimal calcium and copper conditions, the productivity reached 0.027 g PHB L^−1^·h^−1^ [100].

## 6. Comparison of the Typical Substrates for PHA Production

A summary of the typical bacterial species’ productivity using the three categories of substrates is given in Table 2. Using glucose as a carbon source, productivity of up to 2.42 g·L^−1^·h^−1^ can be achieved [101]. However, the yields of PHAs are usually low, approximately 0.3–0.5 g/g of glucose. In contrast, plant oils have been shown to provide higher yields, of approximately 0.6–0.8 g/g, as they have higher carbon contents. However, the productivity of PHAs using oils is much lower than using carbohydrates, as can be seen from Table 2. The productivity of PHAs from hydrocarbons is much lower (except octane) compared to carbohydrates and oils, but octane is much more expensive than carbohydrates and oils.

## 7. PHA Production within a Biorefinery

At present, the major carbon sources for commercial PHA production are still food-based glucose and vegetable oils. The use of hydrocarbon from waste plastics is only exploited at the laboratory scale, but more research should be conducted to improve its yields and productivity. The use of waste streams from biorefinery, including glycerol and lignocellulosic sugars, is a promising route for sustainable production of PHAs. So far, no comprehensive cost-effective methods have been developed to fully harness fermentable sugars from lignocellulose. We now propose an integration of PHA production within a biorefinery, which may offset the cost of bioethanol by co-production of value-added PHAs.

A biorefinery is defined as “a facility that integrates biomass conversion processes and equipment to produce fuels, power, and chemicals from biomass” [106]. A biorefinery usually has two routes to “refine” biomass, which results in different relative quantities of its products. The first is a biochemical route via “sugar”. The second is a thermochemical route via “syngas”, in which biomass is gasified into syngas, and the syngas is used to synthesize liquid fuels via the Fischer–Tropsch process. Mixed feedstock, including lignocellulose and agro-industrial wastes, can be processed in a modern biorefinery [107]. This section will discuss the co-production of PHAs based on the “biochemical route” biorefinery.

In a petroleum-based refinery, some of the refined petroleum is transformed into chemical feedstocks, such as ethylene, propylene and terephthalic acid, which are used to synthesize polymers. Most of the conceptual biorefineries produce “biofuels” through refining biomass as the target products. Suitable polymers have not yet been identified as co-products, like in a petroleum-based refinery. Based on current developed technology, PHA could be a suitable candidate in a biorefinery.

**Figure 6:**
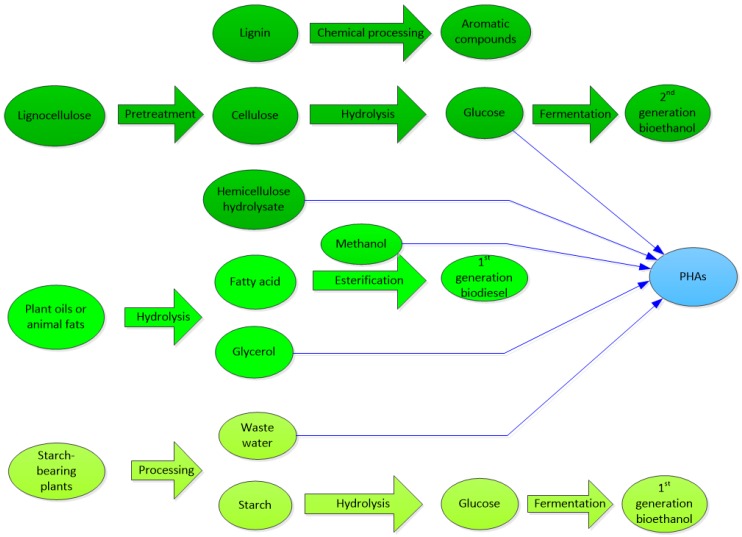
Integration of the production of PHAs into a modern biorefinery from lignocellulose (dark green), plant oils or fats (medium green) and plant starch (pale green)

Figure 6 shows the potential routes as to how PHAs can be co-produced in a biorefinery. In the production of bioethanol using starch-bearing plants, the waste water contains carbohydrates [108,109], which can be used to produce PHAs. In the production of biodiesel using plant oils or animal fats, large amounts of crude glycerol are by-produced [73]. Furthermore, methanol is contained in the waste stream [73]. Both the crude glycerol and the methanol could be used to produce PHAs.

In the long run, the second generation industrial biorefineries will refine lignocellulose, including various agriculture residues, agro-industrial wastes, energy crops and forestry residues. When these lignocellulose residues are refined, a large amount of hemicellulose hydrolysate will be produced in the pretreatment process. This hydrolysate will contain a high concentration of simple sugars, which can be fermented to PHAs [49,54,110,111]. Further optimisation of fermentation conditions is needed to improve the productivity. 

**Figure 7:**
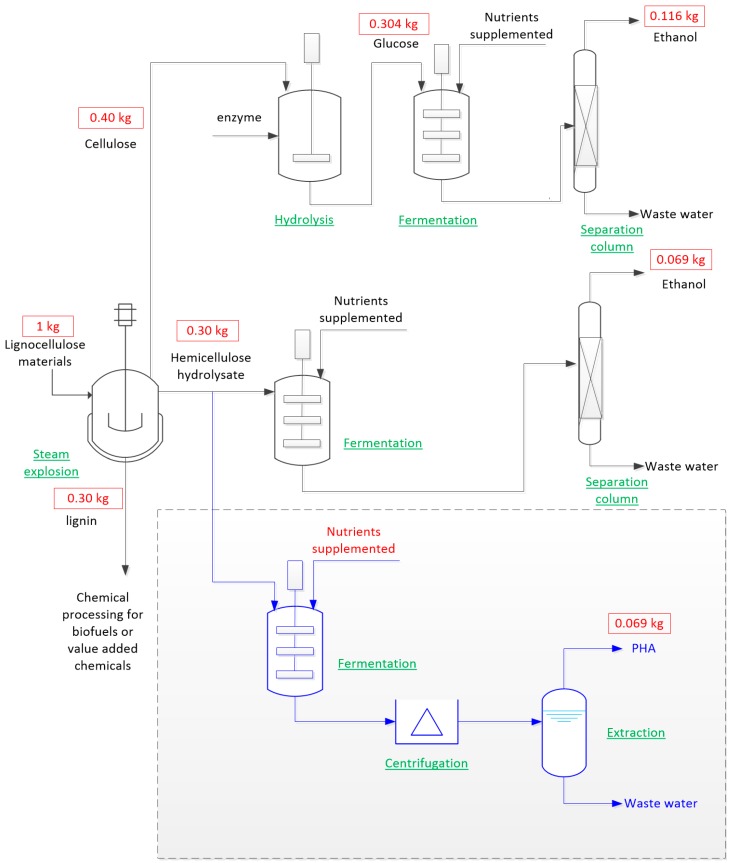
A schematic diagram and mass balance (red) of the proposed integrated biorefinery for producing bioethanol (black) and biopolymer (blue) by various process stages (green). The part in the dashed line box is the proposed production of PHAs using the hemicellulose hydrolysate stream. Cellulose to glucose conversion and recovery efficiency is 0.76 g/g, and fermentation of glucose to ethanol is 0.38 g/g [114]. Hemicellulose to xylose conversion and recovery efficiency is 0.90 g/g, and fermentation of xylose to ethanol is 0.26 g/g [114]. Fermentation of xylose to PHA is 0.26 g/g [48], and fermentation of hemicellulose hydrolysate is 0.90 × 0.26 = 0.23 g/g, where 0.90 is the hemicellulose to xylose conversion and recovery efficiency, which is the same as the hemicellulose hydrolysate used to produce ethanol.

A schematic diagram for the technical route and mass balance is shown in Figure 7 for the production of PHAs using a hemicellulose hydrolysate stream in a biorefinery of lignocellulosic materials. In the technical route, agricultural waste lignocellulose is pre-treated using the steam-explosion method, a commonly-used pretreatment to separate lignin and hemicellulose from cellulose [43]. In this steam-explosion treatment, sized biomass is treated using high pressure saturated steam (160–260 °C) for several seconds to two minutes, and then, the pressure is reduced rapidly, resulting in the rupture of the rigid fibre structure. The treatment leaves the cellulose part as a solid fraction, with lignin redistributed on the surface of cellulose as a result of depolymerisation and melting [112]. The cellulose is hydrolysed to produce glucose, which is used to produce bioethanol. The hemicellulose is hydrolysed, and C5 sugars are released (mainly xylose) as hydrolysates [112]. Currently, the maximum yield of the fermentation is 0.23 g ethanol/g xylose sugar [113]. The maximum yield of PHA from hemicellulose hydrolysate is 0.26 g/g xylose [48]. Therefore, the hemicellulose hydrolysate from 1 kg of lignocellulosic material can produce approximately 0.069 kg of ethanol; alternatively, it can be used to produce approximately 0.069 kg of PHA. Since the fermentation cost for both products is similar and the price of PHA is much higher than ethanol, the use of hemicellulose hydrolysate stream to produce value-added PHA can offset the cost of the second bioethanol production.

## 8. Conclusions and Outlook

The carbon sources for producing PHAs can be classified into three main categories: carbohydrates, triacylglycerols and hydrocarbons. Built on the previous success in the development of a cultivation strategy and metabolic engineering of PHA-producing microorganisms, more research and development activities have been directed towards the use of sustainable feedstocks to alleviate the reliance on food-based feedstocks.

Biorefineries are an emerging concept for the large-scale lignocellulosic biomass to produce biofuels and commodity platform chemicals. The biorefinery by-products of hemicellulose hydrolysates, crude glycerol, waste plant oils and low-grade biodiesels will be potentially sustainable substrates for PHA production, although more research is needed to identify high productivity strains and optimal growth conditions to improve the yields and productivity of PHAs for efficient conversion.

Integration of PHA production with biorefineries will open a new avenue towards producing bioplastics and offset the high price of the second generation bioethanol and biodiesel. A rough estimate indicates the techno-economical feasibility of the integration. By putting PHA production into the framework of a biorefinery, it will enable the biorefinery industry to produce both novel biopolymers and biofuels, like the modern petroleum-based petrochemical industry.

## Figures and Tables

**Table 1:** Main polyhydroxyalkanoate (PHA) producers and their substrate.

Company	Main Substrate	Main Product (Brand)	Production Capacity (Tons/Year)
Biomer (Krailling, Germany)	Sucrose [8]	P3HB	Not available
Bio-on (Bologna, Italy)	Beet sugar [9]	MINERV^®^-PHA	10,000
Tianjin Green Bio (Tianjin, China)	Sucrose [8]	P3HB	10,000
Kaneka (Takasago, Japan)	Vegetable oil [8]	PHBH	1000
Biocycle PHA Industrial (Serrano, Brazil)	Cane sugar [10]	P3HB	100
Tian’an (Ningbo, China)	Corn [8]	P3HB	10,000
Metabolix (Woburn, MA, USA)	Corn [11]	Mirel™-PHA	50,000

PHA = Polyhydroxyalkanoate, P3HB = Poly-3-hydroxybutyrate, PHBH = poly-3-hydroxybutyrate-co-3-hydroxyhexanoate.

**Table 2:** Comparison of the three categories of carbon sources for their productivity and yields of PHAs. All of the data are from bench scale or large-scale fermentors.

Feedstock	Strains	Cell Dry Weight (g/L) ^a^	PHA Content (%) ^b^	Type of the PHA	Productivity (g·L^−1^·h^−1^) ^c^	Yield (g PHA/g Substrate)	Reference
*Carbohydrates*							
* Glucose	*R. eutropha*	164	73.8	P3HB	2.42	0.32–0.48	[7] **
* Glucose + lauric acid	*A. hydrophila*	50	50.0	P(3HB-co-3HHx)	0.54	-	[102] **
Xylose	*P. cepacia*	2.6	60	P3HB	-	0.11	[45]
* Sucrose	*A. latus*	143	50	P3HB	3.97	0.40	[103]
* Molasses	*A. vinelandii*	38.3	60	P3HB	1.4	0.12	[29]
Lignocellulose hydrolysates	Various micro-organisms	1–145	32–89	P3HB	0.3–105	0.11–0.40	[104]
Whey	*E. coli* harbouring *A. latus* genes	119.5	80.2	P3HB	2.57	0.52	[32]
*Triacylglycerols*							
* Plant oils	*R. eutropha*	120	62.5	P3HB	0.96	0.72–0.76	[66] **
Waste frying oil	*R. eutropha*	10.4	36.5	P3HB	0.14	0.19–0.34	[86]
Glycerol	*P. cepacia*	23.6	31.4	P3HB	0.6-1.5	0.062	[76]
Methanol	*M. organophilum*	250	52.0	P3HB	1.8-2.0	0.19	[81]
*Hydrocarbons*							
*n*-Octane	*P. oleovorans*	37.1	32.6	mcl-PHA	0.25	-	[105]
Polystyrene oil	*P. putida*	1	25.0	mcl-PHA	0.015	0.10	[94]
Methane	*Methylotroph* spp.	1.5	51.0	P3HB	0.031	0.55	[99]

* Commercially-used carbon source; ** large-scale data; ^a^ cell dry weight (CDW) is the mass of dry cells in a bioreactor; ^b^ PHA content is the percentage of mass of PHA in dry mass of cells; ^c^ productivity is calculated by the following formula: productivity = PHA content/time of fermentationan important parameter for measuring the efficiency of a fermentation process. Hyphen indicated no data available.
